# A Rare Case of Postoperative Pneumatosis Intestinalis Complicated by Sigmoid Colon Perforation Requiring Immediate Surgical Intervention

**DOI:** 10.7759/cureus.78480

**Published:** 2025-02-04

**Authors:** Bola Habeb, Sandy Khair, Matthew Fowler

**Affiliations:** 1 Internal Medicine, University of Florida College of Medicine, Ascension Sacred Heart, Pensacola, USA; 2 Radiation Oncology, Cairo University, National Cancer Institute, Cairo, EGY

**Keywords:** benign pneumatosis intestinalis, bowel wall gas, incidence rate of pneumatosis intestinalis, life-threatening pneumatosis intestinalis, non-surgical pneumatosis intestinalis, pneumatosis cystoides intestinalis (pci), surgical management of pneumatosis intestinalis

## Abstract

Pneumatosis intestinalis (PI) is a rare but potentially serious gastrointestinal condition with diverse clinical presentations. Pneumatosis intestinalis is identified by the presence of gas in the extraluminal intestinal wall and is further subclassified into benign pneumatosis intestinalis (BPI) and life-threatening pneumatosis intestinalis (LTPI). Prompt and accurate diagnosis, often aided by imaging studies, is crucial to guide appropriate management. Treatment strategies vary depending on the severity of symptoms and underlying etiologies. Collaboration between gastroenterology, radiology, and general surgery is essential to optimize patient outcomes in cases of PI. Herein, we present a case of PI complicated with intestinal perforation that was managed surgically.

## Introduction

Pneumatosis intestinalis (PI), also known as pneumatosis cystoides intestinalis (PCI), is a rare medical condition characterized by multiple gas-filled pockets within the wall of the intestines. These cysts are typically filled with gas, such as oxygen or nitrogen, and can be seen in imaging studies like X-rays, CT scans, or ultrasound. Pneumatosis intestinalis is a radiological finding rather than a diagnosis, indicating an underlying condition. Pneumatosis intestinalis is subclassified based on the etiology into primary (idiopathic) PI that can occur spontaneously without any identifiable cause. Secondary PI is likely associated with an identifiable underlying medical condition, trauma, or medical procedure [1]. It can also be subclassified by disease severity into benign and life-threatening PI.

Determining the prevalence of PI is challenging, as most patients are asymptomatic. A previous study reported an incidence rate of 0.03% in the general population, with a male-to-female ratio of 2.4:1 [2,3]. Pneumatosis intestinalis lesions are primarily located in the colon (46%) and small intestine (27%), with less frequent involvement of both the large and small intestines (7%) and the stomach (5%) [4].

The most common symptoms in patients with benign intestinal pneumatosis are abdominal pain, vomiting, diarrhea, and weight loss. On the other hand, the worrisome features of large intestinal pneumatosis include severe abdominal pain, abdominal distension, diarrhea, and hematochezia. Management should be tailored to the patient’s overall condition and the etiology of PI. In benign PI, management is conservative; however, life-threatening cases usually require urgent surgical intervention [3]. Collaboration between medical, surgical, and critical care teams is essential for proper early diagnosis and management.

This article was previously posted to the Research Square preprint server on November 15, 2024. 

## Case presentation

A 60-year-old male patient with a medical history significant for hypertension, hyperlipidemia, and degenerative spinal disease presented to the hospital due to abdominal discomfort. Two weeks prior, he underwent lumbar spine fixation surgery with an anterior approach, and shortly thereafter he developed watery diarrhea. Three days before his presentation, he experienced steadily worsening abdominal distention, abdominal discomfort, and nausea. The patient described his abdominal discomfort as diffuse, seven out of ten in severity, with cramping abdominal pain localized more to the left lower quadrant. Pain was continuous, non-radiating, worsened with movement, and somewhat improved with rest. He denied any fever, chills, or myalgias. A review of systems was otherwise unremarkable.

Physical examination revealed a temperature of 37.2°C, blood pressure of 132/68 mmHg, pulse of 72 beats per minute (bpm), 18 breaths per minute, and oxygen saturation of 99% on ambient air. He appeared nontoxic and in no acute distress. His abdominal exam revealed a midline vertical incision with appropriate wound healing, otherwise soft with diffuse tenderness to deep palpation. Normal cardiovascular exam with a regular rate, rhythm, and no audible murmurs. Pulmonary examination demonstrated clear lungs to auscultation with no wheezing, rales, or rhonchi.

Admission laboratory data are demonstrated in (Table 1).

**Table 1:** Laboratory data obtained on admission BUN: blood urea nitrogen; AST: aspartate aminotransferase; ALT: alanine aminotransferase

Parameters	Reference range, adults	Patient's values on admission
Hemoglobin (g/dL)	12.0–15.5	14.4
Hematocrit (%)	34.9–44.5	42.5
White cell count (per mm^3^)	3500–10500	11900
Platelet count (per mm^3^)	150,000–450,000	468,000
Sodium (mEq/dL)	135–145	139
Potassium (mEq/dL)	3.5–5.1	3
Bicarbonate (mEq/dL)	22–29	23
BUN (mg/dL)	12–21	27
Creatinine (mg/dL)	0.7–1.2	1.74
Ionized calcium (mg/dL)	4.7–5.4	4.6
Lactate (mmol/L)	0.9–1.7	1.3
Alkaline phosphatase (units/L)	55–142	98
AST (units/L)	12–31	22
ALT (units/L)	9–29	30
Total bilirubin (mg/dL)	0.1–1.1	1.4
Direct bilirubin (mg/dL)	0.0–0.3	0.1

Imaging studies

**Figure 1:**
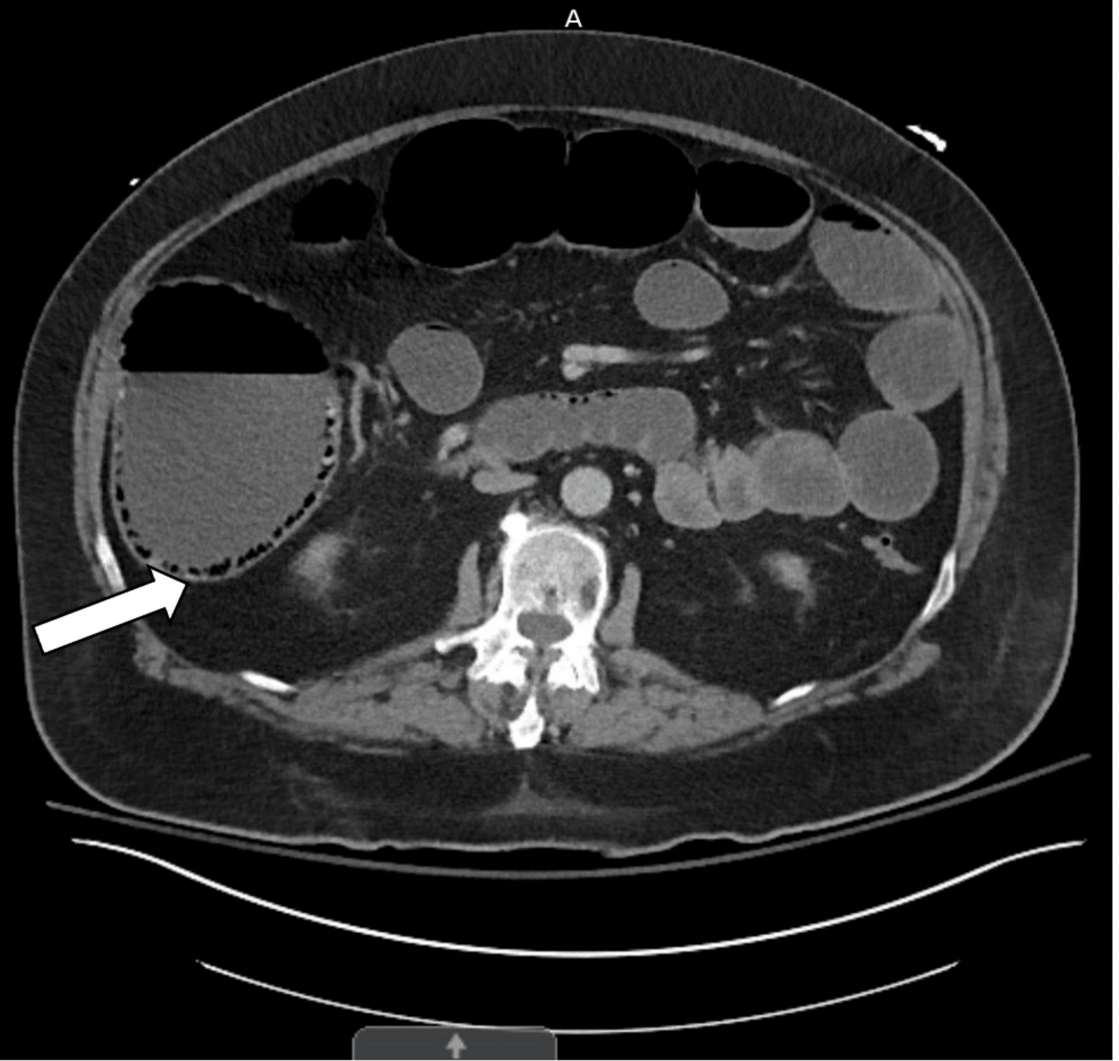
A CT scan of the abdomen and pelvis with IV contrast showed dilated ascending colon with circumferential pneumatosis intestinalis (arrow).

A computed tomography (CT) scan of the abdomen and pelvis with intravenous (IV) contrast showed colonic obstruction with the diameter of the distended ascending colon approaching 11 cm with circumferential PI present (Figure 1).

**Figure 2:**
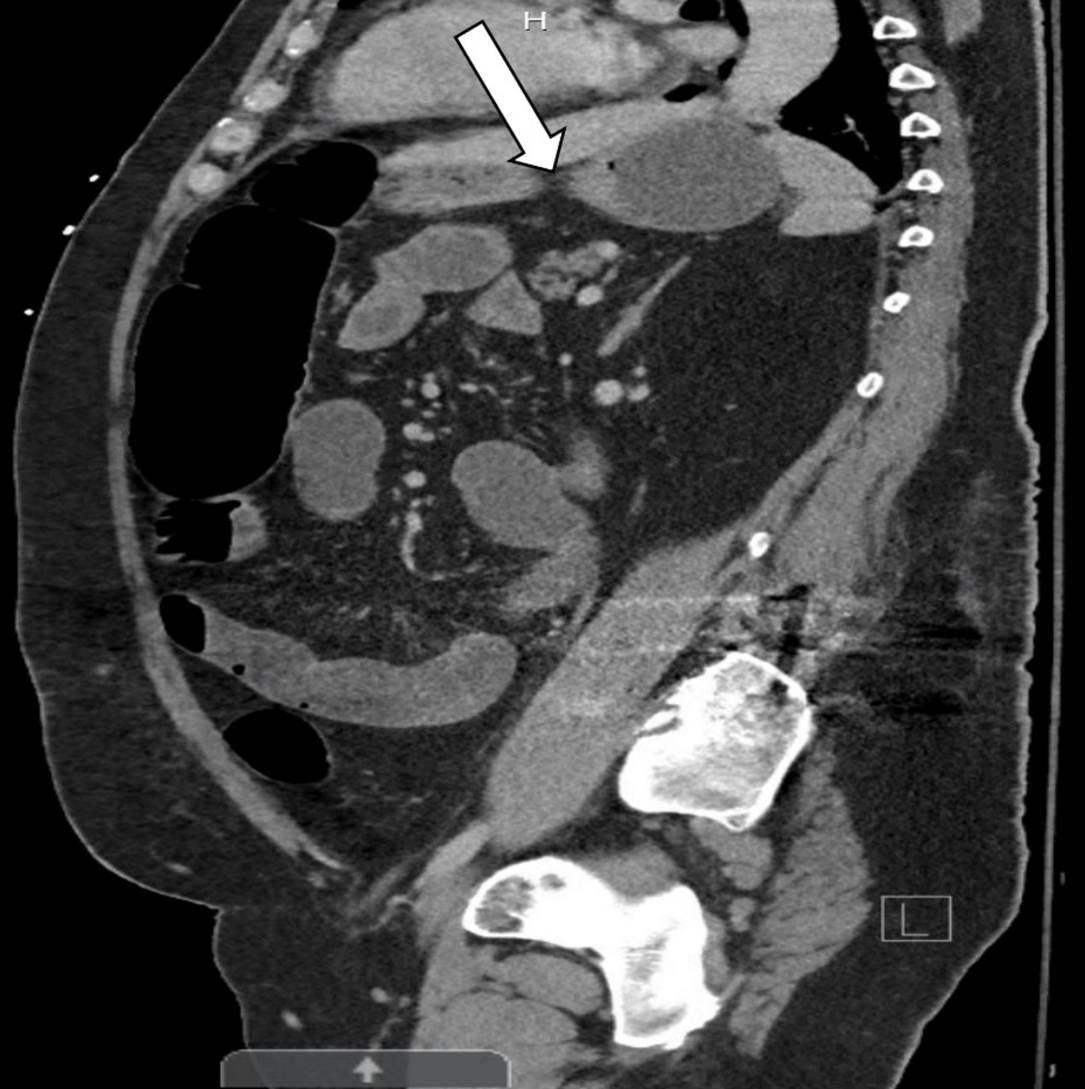
A CT scan of the abdomen and pelvis showed that the transition point was in the mid-descending colon (arrow).

The transverse colon was also distended with pneumatosis, while the descending colon was less distended; a transition point was present in the mid-descending colon without any observed mass (Figure 2).

Hospital course

**Figure 3:**
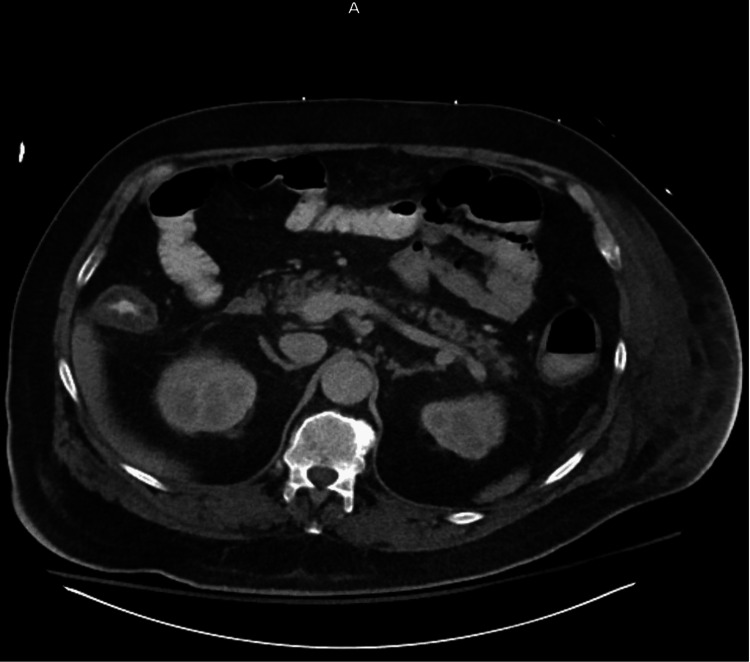
A follow-up CT scan of the abdomen and pelvis showed a normal-sized intestine with no pneumatosis intestinalis.

Intravenous piperacillin/tazobactam was initiated for empiric antibiotic coverage along with IV fluids and hyperbaric oxygen. A nasogastric tube was inserted and placed on low-intermittent suction, and the patient was kept nil per os (NPO) for bowel rest. Given the CT findings indicating a transition point and concern for intestinal obstruction, the patient underwent flexible sigmoidoscopy. The procedure revealed mucosal swelling, erosions, and a substantial amount of liquid stool, but there were no signs of intrinsic obstruction, diverticular disease, or inflammatory bowel conditions such as Crohn's disease or ulcerative colitis. A rectal tube was inserted for colonic decompression, which initially improved the patient's symptoms so that he was able to pass gas. On the following day, he developed severe abdominal pain and sepsis with a temperature of 39.6°C, sinus tachycardia up to 130 bpm, and worsening leukocytosis from 7.8 to 15.4 K/ul. Out of concern for acute bowel perforation, the patient underwent emergent exploratory laparotomy that confirmed a 0.5 cm sigmoid perforation that was oversewn with the placement of an epiploic Graham patch for definitive repair. The postoperative course demonstrated an improvement in the patient's symptoms. A repeat CT abdomen/pelvis with IV contrast performed on postsurgical day five showed expected postsurgical changes related to exploratory laparoscopic Graham patch repair with no evidence of leak into the peritoneal cavity, moderate colonic wall thickening, and resolution of the PI (Figure 3).

The patient completed a 10-day course of IV piperacillin/tazobactam,s and his general condition improved. Outpatient follow-up revealed a stable recovery course.

## Discussion

Pneumatosis intestinalis is identified by the radiographic detection of air within the bowel wall. The pathogenesis of PI is considered multifactorial and yet to be clearly understood. Some PI cases are incidental findings associated with a benign presentation, while others manifest as life-threatening conditions.

Pneumatosis intestinalis can occur in a variety of pathologies and result from numerous underlying causes. It may occur as a postoperative complication following abdominal surgeries, particularly due to intestinal manipulation. Gastrointestinal diseases such as inflammatory bowel disease (including Crohn's disease and ulcerative colitis), diverticulitis, and gastrointestinal infections are also associated with PI. Ischemic bowel disease, resulting from reduced blood flow to the intestines, is another significant cause. Additionally, bowel obstruction can lead to the accumulation of air within the bowel wall. In the context of transplantation, PI may develop during the postoperative period following intra-abdominal organ transplants. Rarely, PI can be seen in individuals receiving mechanical ventilation [5]. The sequelae can range from benign to life-threatening, and hence, management can be equally varied, from simple outpatient observation to emergent surgical intervention. Pneumatosis intestinalis's variety of presentations highlights the importance of careful physical examination and clinical correlation to the radiographic findings to ensure accurate diagnosis and appropriate management. 

Multiple studies conducted by the Eastern Association for the Surgery of Trauma (EAST), the Memorial Sloan-Kettering Cancer Center, and Mount Sinai Medical Center have collectively highlighted common indicators for surgical intervention in PI, such as hypotension, acute abdomen, elevated lactate, low bicarbonate, high white cell count, age ≥60, acute renal impairment, and CT scan findings like bowel ischemia, obstruction, or perforation [6].

**Figure 4:**
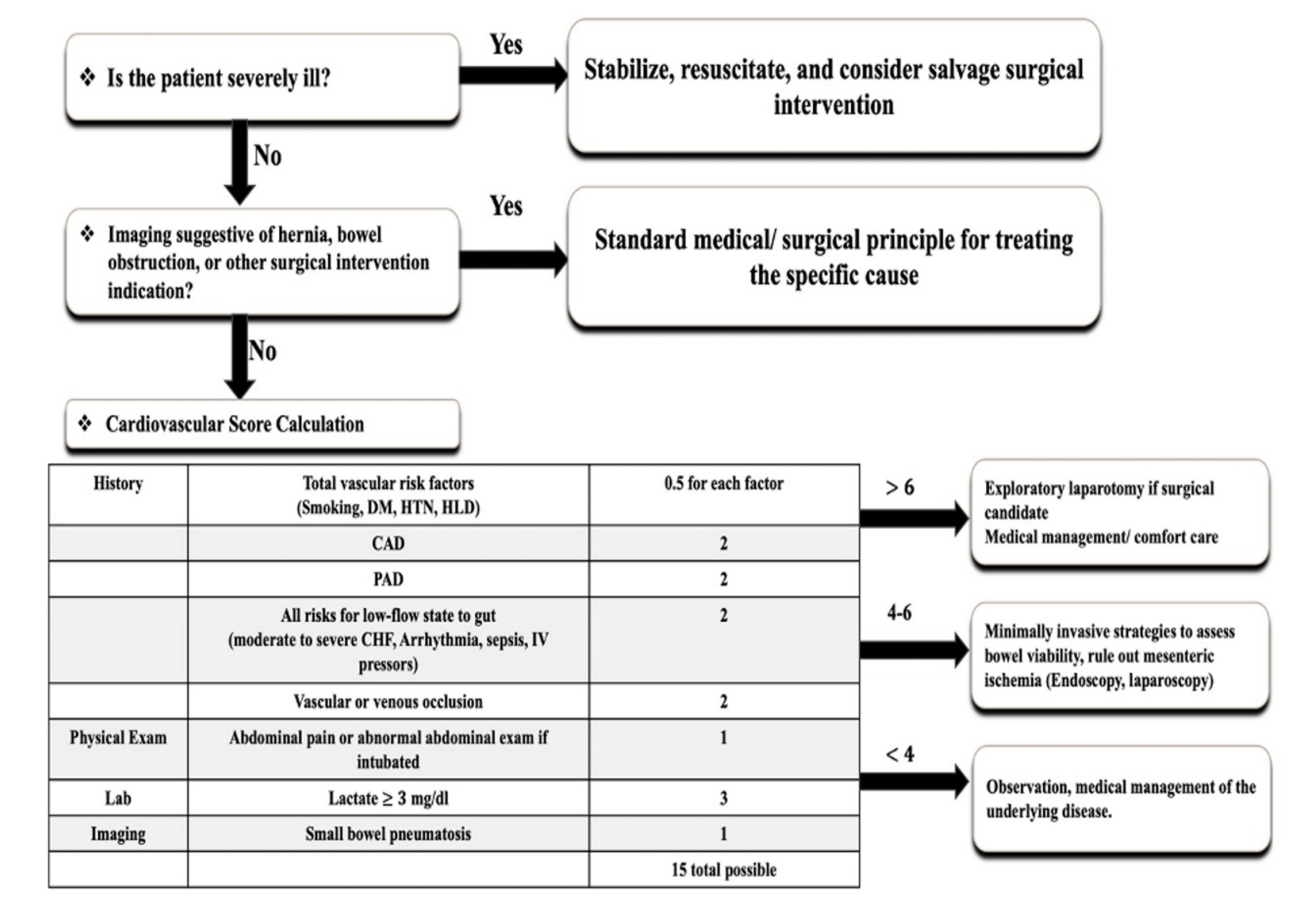
Management algorithm of pneumatosis intestinalis DM: diabetes mellitus; HTN: hypertension; HLD: hyperlipidemia; CAD: coronary artery disease; PAD: peripheral artery disease; CHF: congestive heart failure This image has been adapted from reference [7] which has been adapted from the Journal of Gastrointestinal Surgery; Volume 14, Wayne E, Ough M, Wu A, Liao J, Andresen KJ, Kuehn D, and Wilkinson N, Management Algorithm for Pneumatosis Intestinalis and Portal Venous Gas: Treatment and Outcome of 88 Consecutive Cases; Pages 437-48; Copyright © 2010 Society for Surgery of the Alimentary Tract. Published by Elsevier Inc.

A management algorithm has been proposed to guide clinical decision-making based on a numeric score. The algorithm begins by assessing whether the patient is severely ill. If so, the priority is to stabilize, resuscitate, and consider salvaging surgical intervention. If the patient is not severely ill, imaging is evaluated for signs of hernia, bowel obstruction, or other surgical indications. If such findings are present, standard medical or surgical principles are applied to treat the specific cause. If imaging does not indicate a surgical condition, a cardiovascular risk score is calculated. This score is based on the presence of vascular risk factors, including smoking, diabetes mellitus (DM), hypertension (HTN), and hyperlipidemia (HLD) (0.5 points each); coronary artery disease (CAD) (two points); peripheral artery disease (PAD) (two points); low-flow states (severe congestive heart failure, sepsis, or IV pressors) (two points); physical exam findings (severe abdominal pain or abnormal findings in intubated patients) (one point); laboratory results (lactate ≥3 mg/dL) (three points); and imaging findings suggestive of bowel pneumatosis (one point). A total score guides management: scores greater than six suggest exploratory laparotomy or comfort care; scores of four to six indicate minimally invasive approaches to assess bowel viability; and scores <4 favor observation and medical management of the underlying disease (Figure 4) [7].

Hyperbaric oxygen therapy has also been found to be effective when given for three days to relieve persistent symptoms, owing to its ability to enhance tissue oxygenation, promote tissue repair, and possibly decrease gas buildup in the intestinal wall [8].

In our case, the patient initially presented with a score of two, consisting of 0.5 points each for HTN and HLD and one point for imaging findings suggestive of bowel pneumatosis. Based on this evaluation, conservative management was initiated, including bowel rest, IV antibiotics, IV fluids, and hyperbaric oxygen. However, the patient's score subsequently worsened to five, with two points attributed to HTN, HLD, and bowel pneumatosis as previously described, along with one point for severe abdominal pain and two points for sepsis. This clinical deterioration necessitated surgical intervention to identify and address the underlying cause.

In summary, asymptomatic patients do not need to be treated; however, if an underlying medical condition contributes to PI, that condition must be addressed. Patients with mild symptoms can be treated by using antibiotics with anaerobic coverage and an elemental diet. In addition to the above, patients with moderate to severe symptoms may require endoscopy or laparotomy if still symptomatic despite medical treatment or if they show clinical signs of deterioration or medical instability.

## Conclusions

Individuals diagnosed with PI must work closely with their healthcare providers to determine the underlying cause and develop an appropriate treatment plan. The prognosis and outcomes can vary widely based on the specific circumstances of each case. Benign PI is identified in the absence of alarming features such as portal venous gas, decreased bowel wall enhancement, and bowel dilatation. These benign cases can be managed conservatively with close observation in case complications occur. On the other hand, life-threatening pneumatosis intestinalis (LTPI) with worrisome features should be urgently managed with surgical intervention.
